# Association of *Blastocystis species* subtypes 3 and 4 with intestinal inflammation and response to metronidazole in symptomatic patients

**DOI:** 10.1186/s12879-026-13047-6

**Published:** 2026-04-01

**Authors:** Noha M. Amin, Amira R. Ismail, Shimaa M. Abdel Aal, Aly ELkazaz, Hala Ramadan, Ahmed Mohamed Shenawy, Shaimaa Mohamed Ali, Shimaa Saad El-Din, Magda SA Abdeltawab

**Affiliations:** 1 Department of Medical Parasitology, Faculty of Medicine, Cairo University, Cairo, Egypt; 2 Department of Pediatrics, Faculty of Medicine, Cairo University, Cairo, Egypt; 3 Department of Internal medicine, Faculty of Medicine, Cairo University, Cairo, Egypt; 4 Department of Medical Biochemistry and Molecular Biology, Faculty of Medicine, Cairo University, Cairo, Egypt; 5 Department of Medical Biochemistry and Molecular Biology, Faculty of Medicine, Badya University, Giza, Egypt

**Keywords:** *Blastocystis spp.*, Fecal calprotectin, TNF-α, RFLP, BC subtypes

## Abstract

**Background:**

*Blastocystis spp.* (BC) often passes as a harmless resident of the gut. However, it may be associated with gastrointestinal disturbances, such as abdominal pain and diarrhea. The variation in the clinical spectrum of BC infection is, in part, due to its diverse genotypic profile, where ongoing research continues to discover new BC subtypes. This study aimed at investigating the association between different BC subtypes and intestinal inflammation as assessed by fecal calprotectin (FCP) and tumor necrosis factor alpha (TNF-α) levels.

**Methods:**

Patients complaining of gastrointestinal tract (GIT) symptoms were investigated for the presence of BC infection by microscopy and stool culture on Jones’ medium. Out of 163 patients, 88 were confirmed to be infected with BC. They were further divided according to the dominant BC subtype by RFLP genotyping. Patients were treated with metronidazole at a dose of 250 mg 3 times daily for ten days and were evaluated for symptom improvement after the treatment course. Moreover, FCP and TNF-α levels were measured in stool samples by ELISA before and after therapy.

**Results:**

BC subtype 3 was found to be the dominant subtype in 62 samples (70.5%), while subtype 4 was dominant in 26 samples (29.5%). A higher number of patients infected with BC subtype 3 reported the presence of GIT manifestations. After treatment, 17 subtype 3-infected patients (27.4%) witnessed improvement of their GIT symptoms, as compared to 9 patients infected with BC subtype 4 (34.6%). In subtype 3 infected patients, 71% were free of BC stages by both microscopic examination and stool culture, while 92.3% of patients infected with subtype 4 showed fecal parasite clearance. FCP and TNF-α levels were higher in patients infected with BC subtype 3 as compared to BC subtype 4, both before and after treatment with metronidazole. In the BC subtype 3-infected groups, fecal TNF-α and FCP levels decreased significantly after treatment.

**Conclusion:**

In our cohort study, BC subtype 3 was the most prevalent subtype among patients reporting gastrointestinal symptoms. The ST3 group exhibited numerically higher inflammatory markers compared to ST4. These trends shed light on the potential link between *Blastocystis* genetic diversity and intestinal inflammation. Although the observational nature of this study and the unequal group sizes limit the ability to establish a definitive causal relationship, these findings provide a BC subtype-oriented diagnostic and therapeutic design within a hospital-based setting that could facilitate the design of more tailored management strategies for patients with BC-associated symptoms.

**Graphical Abstract:**

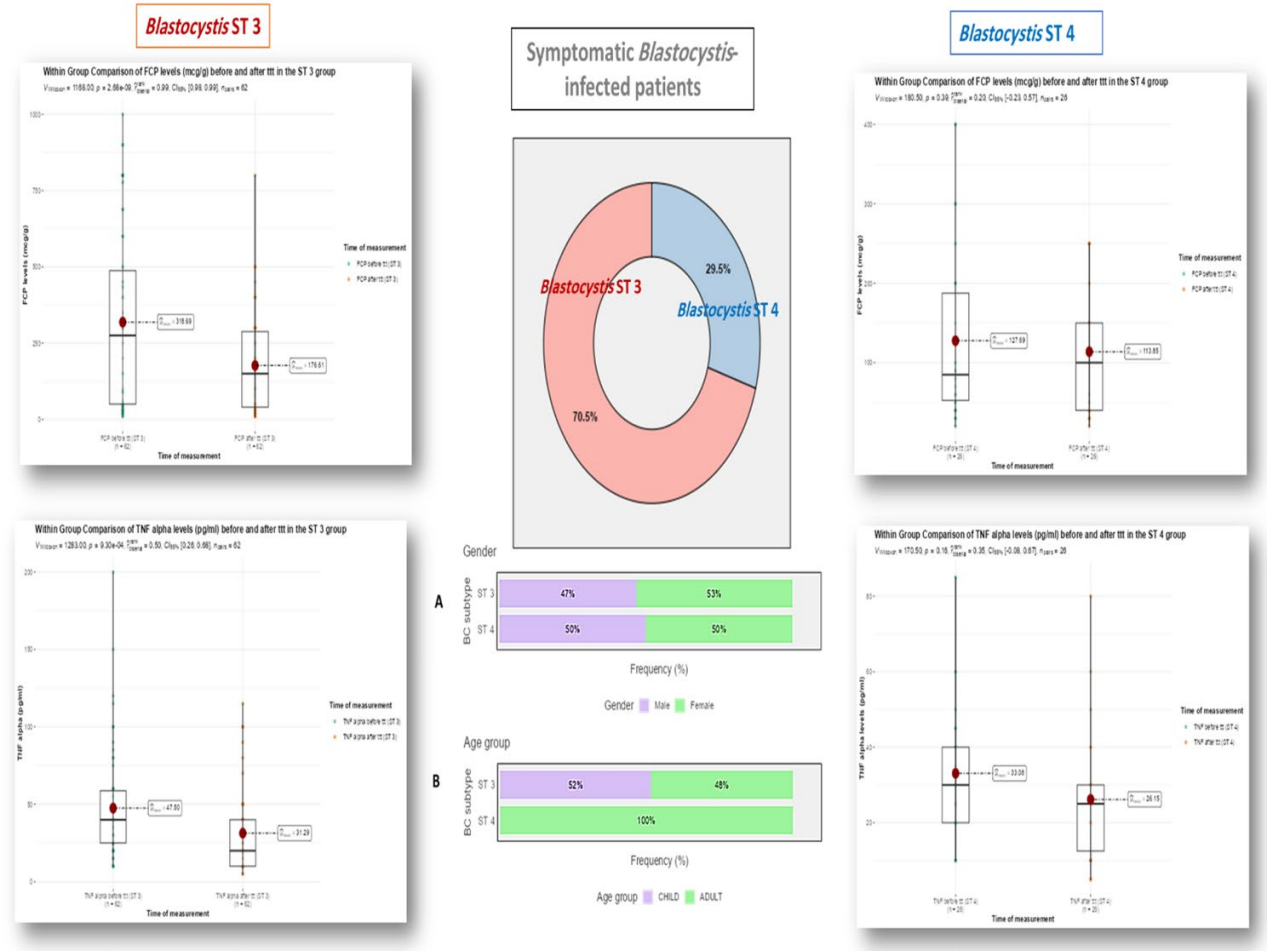

**Supplementary Information:**

The online version contains supplementary material available at 10.1186/s12879-026-13047-6.

## Background

*Blastocystis spp.* (BC) is a controversial parasite as regards its classification, pathogenicity, and association with various gastrointestinal, and even systemic diseases [1]. Since its identification at the beginning of the 19th century, it was regarded as a harmless commensal, or a causative of asymptomatic infection. However, studies have emerged linking BC to symptomatic gastrointestinal tract (GIT) infection, in addition to other GIT disorders such as inflammatory bowel disease, Crohn’s disease and ulcerative colitis [2, 3]. *Blastocystis spp.* is conventionally treated with metronidazole, for which varying degrees of efficacy in terms of clinical recovery and parasite eradication have been reported. Resistance to metronidazole is a major challenge in the management of blastocystosis and is complicated by the discrepancy between the degree of resistance among different BC subtypes [4]. Analysis of small subunit ribosomal RNA has identified 44 BC subtypes in both human and animal hosts. Subtypes 1–4 have been most frequently isolated from human samples. Different BC subtypes produce different effects on the resident gut microbiota and the local intestinal inflammatory response, which might explain the diverse spectrum of symptomatology among BC infected patients [5, 6].

BC is also diverse in its interactive crossroads with the host immune response. Activation of signal transduction pathways such as Toll-like receptor (TLR) and nuclear factor kappa B (NFκB) pathways, in addition to the stimulation of TH1 and TH2 responses, are examples for the immunopathological effects of BC infection [7]. BC induces intestinal inflammation and gut barrier disruption by increasing the release of pro-inflammatory cytokines, such as tumor necrosis factor alpha (TNF-α), Il-6, Il-1β, and IL-8 [8]. TNF-α is an important marker of GIT inflammation and is produced mainly by macrophages and monocytes [9]. BC derived exosomes were found to induce the release of TNF-α along with other cytokines in a human leukemia monocytic cell line [10].

IL-8 regulates the movement of granulocytes, particularly neutrophils [11]. Among the plethora of mediators secreted by neutrophils, calprotectin has gained special attention since it constitutes about 60% of the protein content of the neutrophilic cytosol and is released into the blood without requiring de novo synthesis [12, 13]. Since calprotectin is abundant in the cytosol of neutrophils, its release is a marker of neutrophil activation. Upon secretion during intestinal inflammation, calprotectin reaches the local inflammatory site in the GIT to be released and finally excreted in feces [14, 15]. Fecal calprotectin (FCP) has been recognized as an indicator of activity, recovery and recurrence of inflammatory gastrointestinal diseases such as Crohn’s disease, ulcerative colitis and inflammatory bowel disease [16]. Elevated FCP levels have been associated with several parasitic infections such as infection with *Entamoeba histolytica*, *Giardia lamblia* and *Blastocystis sp.* [17].

The design of controlled studies comparing the inflammatory potential of different BC subtypes in a clinical setting are particularly challenging. This comparative observational study aimed to explore the prevalence of BC subtypes in a symptomatic cohort of blastocystosis patients. In addition, we sought to investigate the association between the detected BC subtypes and biomarkers of intestinal inflammation, and to observe changes in these biomarkers following conventional metronidazole therapy. Findings from this real-world clinical investigation can highlight subtype-specific differences and generate hypotheses for future mechanistic and controlled interventional studies.

## Materials and methods

### Ethical statement

The current study was conducted in accordance with the ethical principles of the Declaration of Helsinki (2013) and approved by the ethical committee of the Faculty of Medicine, Cairo University (N.99.2025). All samples were handled and processed according to national regulations and institutional guidelines. Participant confidentiality was protected through the anonymization of data.

### Study design and rationale

This study was designed as a comparative observational study of a symptomatic cohort of patients with confirmed *Blastocystis spp.* infection. We employed a design comparing two naturally occurring subtype groups to examine the association between subtype, clinical presentation and inflammatory markers within a frame of daily clinical practice. A non-infected control group was not included, since our objective was to investigate whether different genotypes lead to different inflammatory outcomes within a symptomatic population rather than to re-evaluate the association between *Blastocystis* and symptomatic disease. This symptomatic-only approach was intentionally designed to determine if variations in fecal biomarkers correlate with specific subtype dominance, rather than solely with the presence or absence of the parasite.

Between-group comparisons were analyzed for associations with symptom prevalence and biomarker levels. Within-group pre- and post-treatment assessments were done using paired tests. All analyses are considered exploratory and associational, intended to describe potential relationships rather than establish causal efficacy, which would require a placebo-controlled trial design.

### Study subjects and treatment with metronidazole

Study participants suffering from GIT symptoms were recruited from patients referred to the Diagnostic Research Unit of the Parasitology department (DRUP) of the Faculty of Medicine, Cairo University. BC infection was diagnosed by microscopic examination and stool culture on Jones’ medium.

Inclusion criteria: Patients complaining of GIT symptoms such as abdominal pain, fever, diarrhea, vomiting, loss of weight and appetite, and confirmed to be infected with BC by microscopy and stool culture.

Exclusion criteria: Subjects receiving anti-parasitic treatment during the preceding 12 months; patients complaining of other parasitic diseases as confirmed by patient history and microscopic examination.

### Parasitological examination

Morning stool samples were examined both directly in wet mount preparations and after formol ether concentration for all study samples. Stool preparations were stained with iodine, trichrome and modified Ziehl-Neelsen stains. A positive identification of BC infection was diagnosed by the detection of the vacuolar forms of the parasite in two or more high power fields. Stool culture for the detection of BC was performed by using Jones’ medium enhanced by 10% horse serum and incubated at 37 °C. Microscopic examination for the identification of BC stages was performed every 24 h for 3 days [18].

### Molecular characterization of BC isolates

The Favor Prep-stool DNA isolation Mini Kit (Favorgen Biotech corporation Ping-Tung 908, Taiwan, Cat. No. FASTI001) was used for DNA extraction according to the manufacturer’s instructions. The PCR assay was conducted using Blas-F (GGA GGT AGT GAC AAT AAA TC) as the forward primer and Blas-R as the reverse primer (ACT AGG AAT TCC TCG TTC ATG). The following constituents were used to optimize the condition of the PCR: Reverse primer (1 µl), forward primer (1 µl), PCR master mix (12.5 µl), template DNA (2.5 µl) and Taq polymerase (0.5 L µl). Distilled water was added to reach a total volume of 25 µl [19].

Genotyping of BC was performed using RFLP technique to gain 1.1 kbp SSU rRNA gene products by the restriction endonuclease Hinfl enzyme (New England Bio Labs Inc., MA, USA) [19]. Ten µl of amplified product was digested by the restriction endonuclease Hinfl (1 µl) in a reaction mixture (25 µl), containing 10X buffer (2.5 µl) and nuclease free water (11.5 µl). The digested DNA products were loaded into wells of a 0.8% ethidium bromide-impregnated agarose gel. UV light was used to visualize the DNA fragments which were then compared to a DNA ladder to determine the BC subtype pattern.

Molecular characterization of *Blastocystis spp.* was done using PCR-RFLP due to its established specificity and reproducibility in identifying the most clinically prevalent subtypes (ST1–ST7). Given the prospective nature of this study and the requirement for high-throughput screening of a large patient cohort, PCR-RFLP provided a practical framework for genotype identification within a hospital-based setting.

### Clinical evaluation of patients and treatment with metronidazole

Clinical history was taken from all patients at the beginning of the study. The presence of GIT manifestations such as diarrhea, vomiting, abdominal pain, flatulence, and constipation was recorded. In addition, the improvement of symptoms after treatment with metronidazole was also evaluated. Patients were given metronidazole orally at a dose of 250 mg 3 times daily for 10 days [20].

### Detection of fecal calprotectin (FCP) and fecal TNF-α by ELISA

FCP was detected by ELISA using the Drg: Hybridxl Calprotectin Kit. A value of more than > 200 µg/g stool was considered positive [21]. Fecal TNF-α levels were measured according to the manufacturer’s instructions (Eagle Biosciences, KR9610, USA) [22].

### Statistical analysis

Normality check was performed by Shapiro-Wilk test. As the quantitative data were not normally distributed (*P* < 0.01), non-parametric tests were employed. Mann-Whitney test was used for the analysis of data between groups. Within group comparison before and after treatment was done using the Wilcoxon rank test. Qualitative data were analyzed by Pearson’s Chi-square test for between group comparisons and the McNemar test for within group comparisons. Correlation between data was performed using the Spearman correlation test. Statistical significance was reported at P value < 0.05. Sample size calculation with 0.05 alpha error, confidence interval of 0.95 and power of the study 0.80 was performed.

## Results

### Patient classification according to BC genotyping

**Fig. 1:**
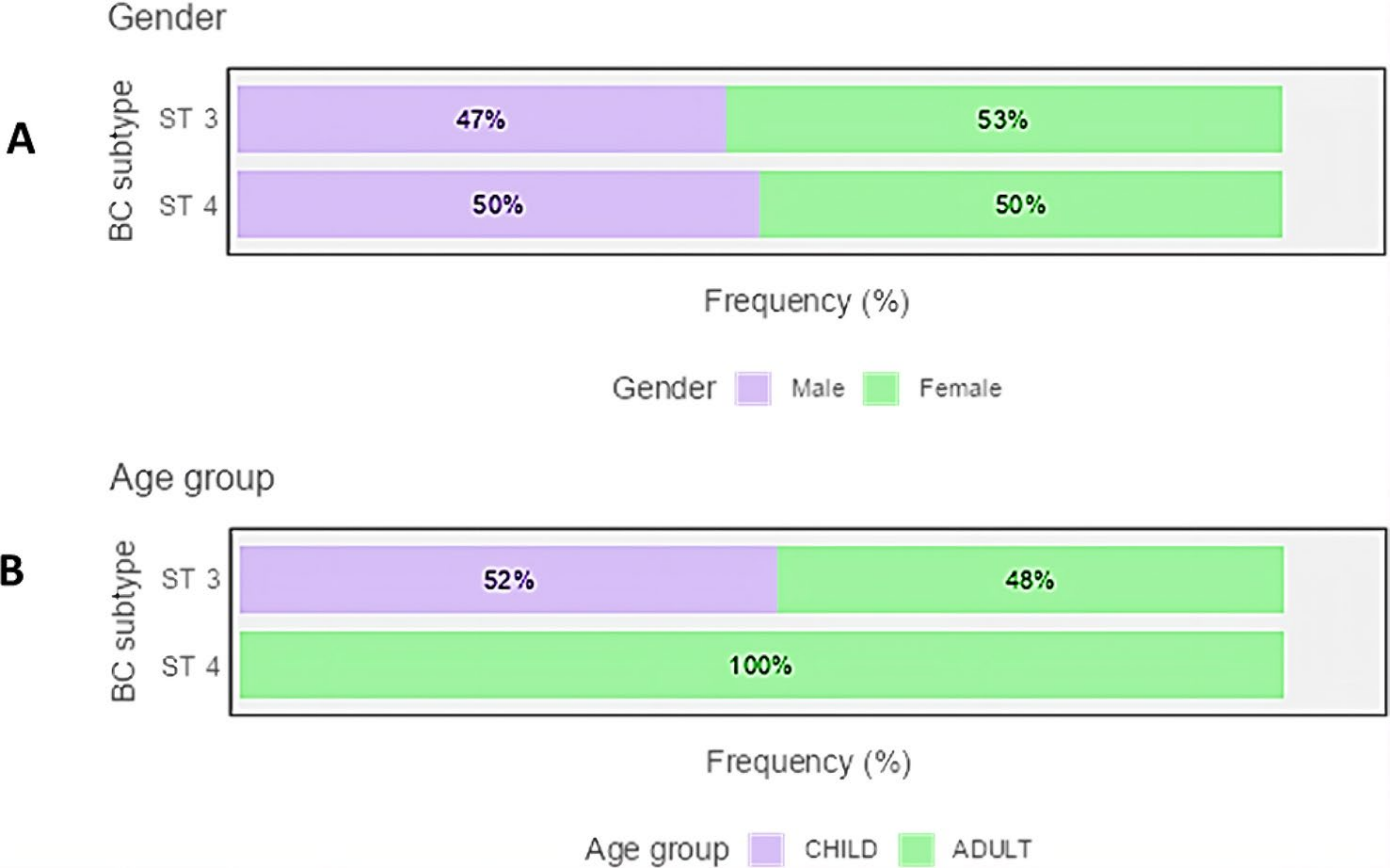
Horizontal stacked bar chart showing percentage distribution of patient gender (**A**) and age group (**B**) among study groups

BC subtype 3 was found to be dominant in 62 samples (70.5%), while subtype 4 was dominant in the remaining 26 samples (29.5%). Age group and sex of study participants are presented in Fig. 1. The mean age of subtype 3 infected patients was 29.2 ± 19.0 years (median = 27.5, IQR = 36.8), while that of subtype 4 infected patients was 35.7 ± 18.2 years (median = 41.5, IQR = 32.5). All patients infected with subtype 4 were adults above the age of 18 years.

### Clinical evaluation of patients

**Fig. 2:**
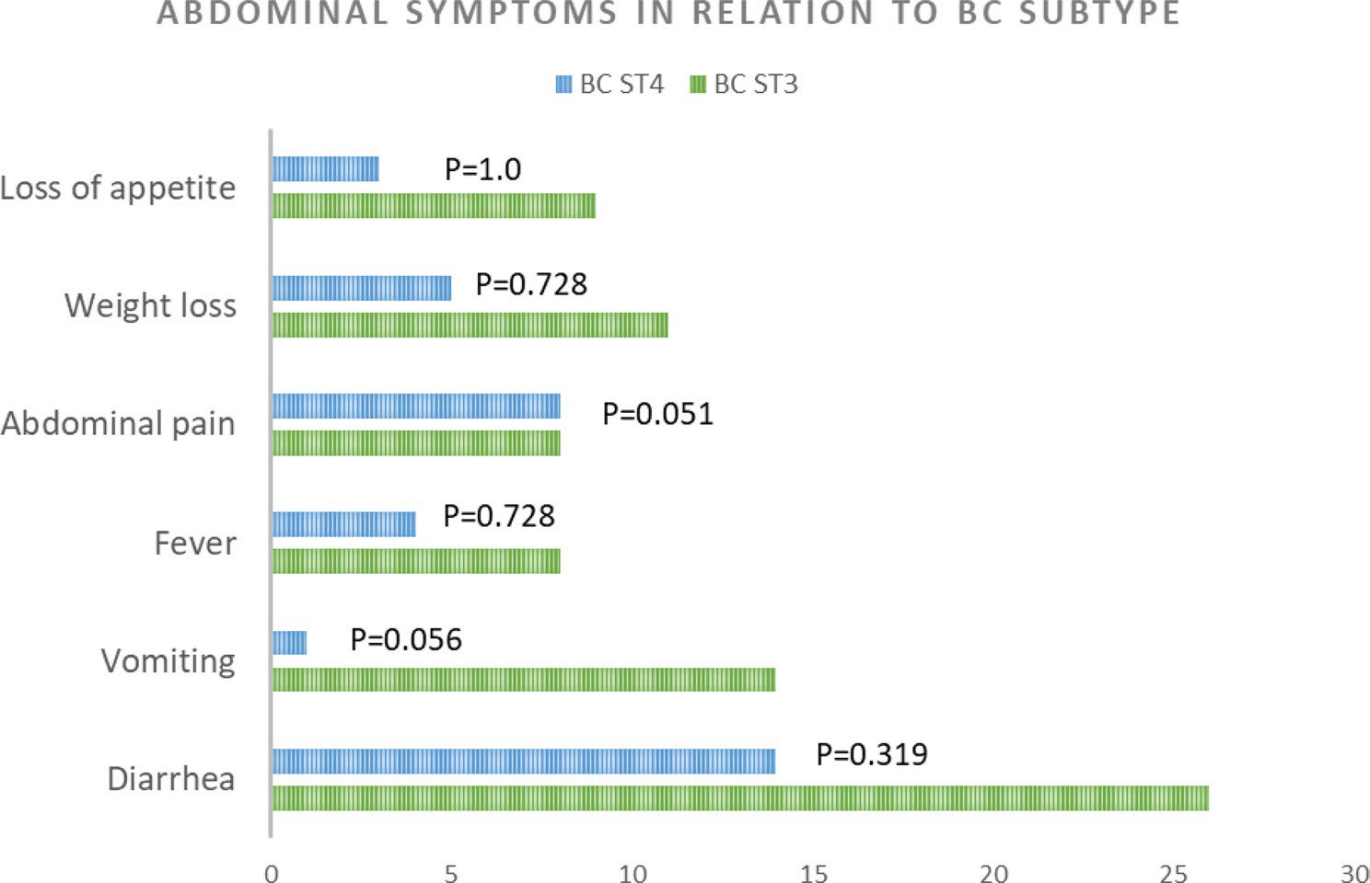
Horizontal column chart representing patient evaluation before treatment with metronidazole. X-axis represents number of patients complaining of various symptoms in reference to the BC subtype in the corresponding stool samples; P value calculated for Fischer’s exact test

Clinical manifestations such as loss of appetite and weight, vomiting, fever and diarrhea were more frequently encountered in BC ST3 patients (Fig. 2). After treatment, 17 patients (27.4%) infected with subtype 3 reported improvement of their GIT symptoms, as compared to 9 patients infected with BC subtype 4 (34.6%). No statistical significance was found between the presence of individual symptoms in each study group, which should be carefully interpreted considering the disparity in sample size between them. In addition, no significant difference was found between the improvement of symptoms in both groups (*P* = 0.61; RR = 0.90, 95% CI [0.66, 1.24]; OR = 0.71, 95% CI [0.27, 1.90]).

### Relation between BC subtype and presence of BC in stool

After treatment with metronidazole, 71% of patients infected with subtype 3 were free of BC stages by both microscopic examination and stool culture. On the other hand, 92.3% of patients infected with subtype 4 showed fecal parasite clearance (Table 1). The rate of parasite persistence (failed clearance) was significantly higher in the ST3 group compared to the ST4 group (*P* = 0.048; RR = 1.39, 95% CI [1.11, 1.75]; OR = 4.91, (95% CI [1.05, 23.0]).

**Table 1:** Number and percentage of samples positive for BC stages by microscopic examination and stool culture in relation to the BC subtype after treatment with metronidazole

After treatment with metronidazole				
Detection of BC in stool samples by microscopy and culture	BC subtype (number of patients; %)		Total	*P* value
	Subtype 3	Subtype 4		
*Detected*	18 (29.0%)	2 (7.7%)	20 (22.7%)	0.048^*^
*Not detected*	44 (71.0%)	24 (92.3%)	68 (77.3%)	
*Total*	62 (70.5%)	26 (29.5%)	88 (100%)	

Fischer’s exact test

*Statistical significance at *P* < 0.05

### Relation between BC genotypes and FCP and fecal TNF-α levels

**Fig. 3:**
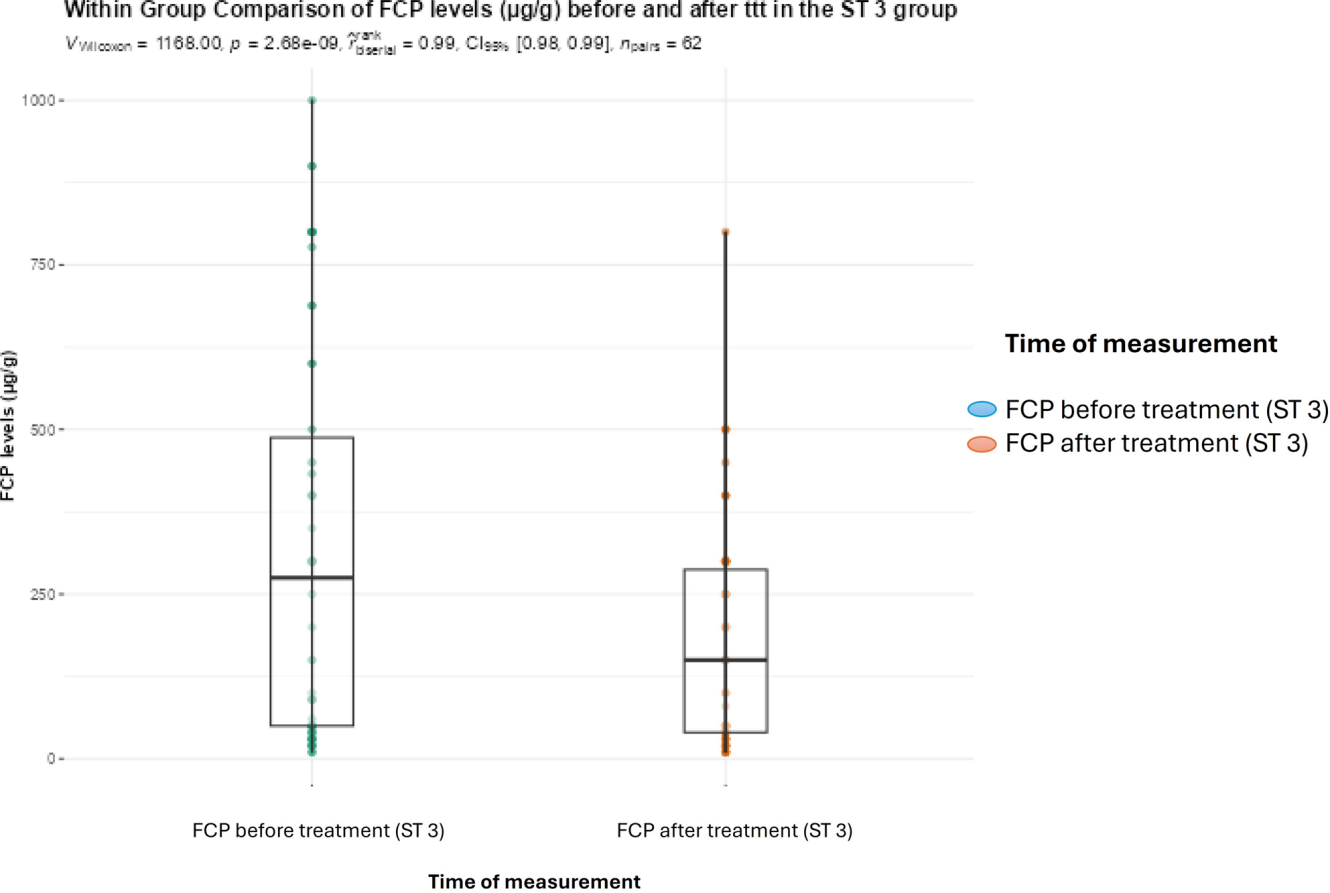
Boxplot showing within group comparison between FCP levels in µg/g (medians and IQRs) before and after treatment with metronidazole in the BC subtype 3 infected group (Wilcoxon rank test; statistical significance at *P* < 0.05)

**Fig. 4:**
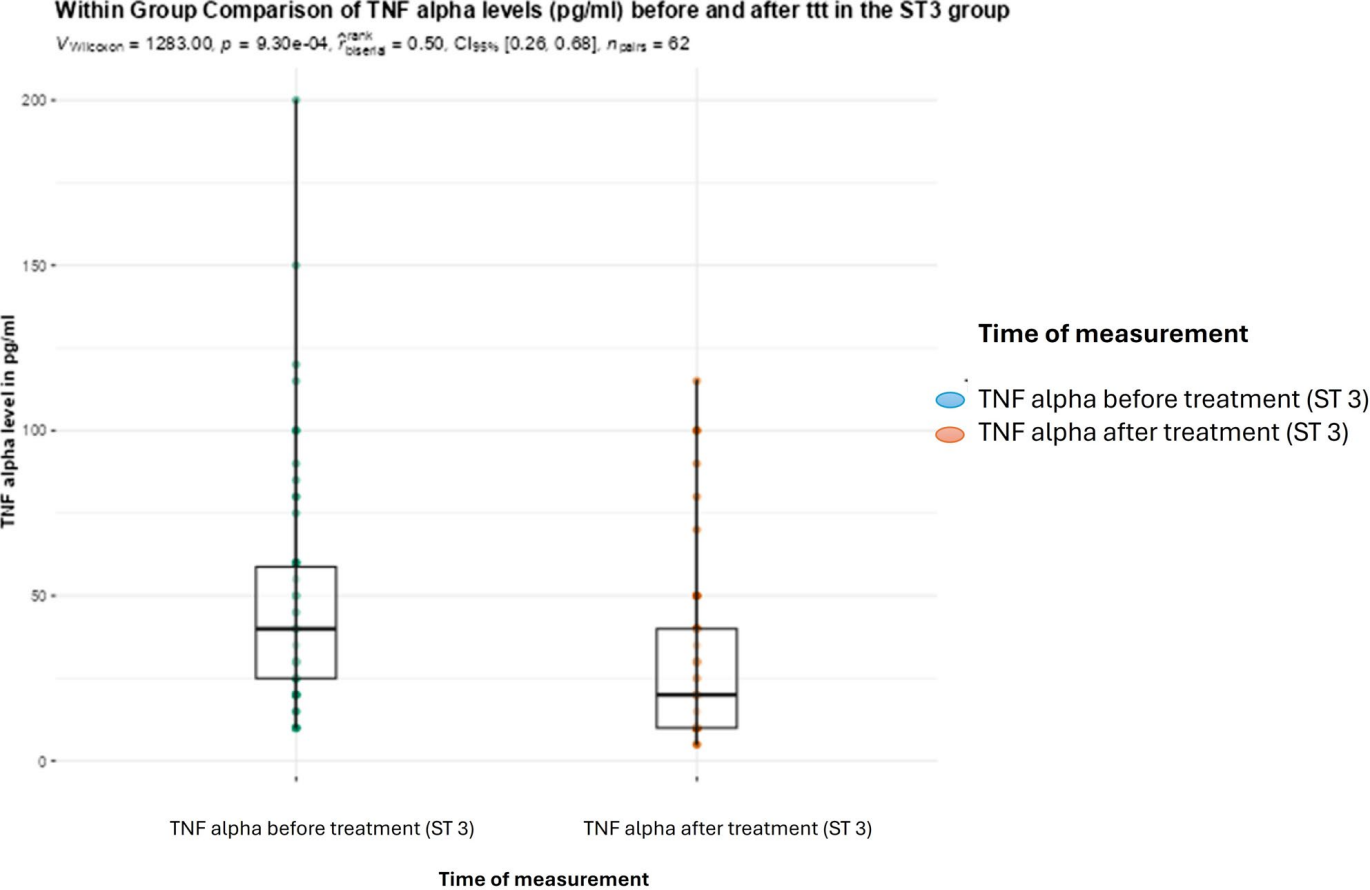
Boxplot showing within group comparison between TNF-α levels in pg/ml (medians and IQRs) before and after treatment with metronidazole in the BC subtype 3 infected group (Wilcoxon rank test; statistical significance at *P* < 0.05)

Within group comparison between FCP levels before and after treatment revealed a significant reduction after the administration of metronidazole in the BC subtype 3 and group (*P* < 0.001). This was also observed for TNF-α levels (Figs. 3 and 4).

**Fig. 5:**
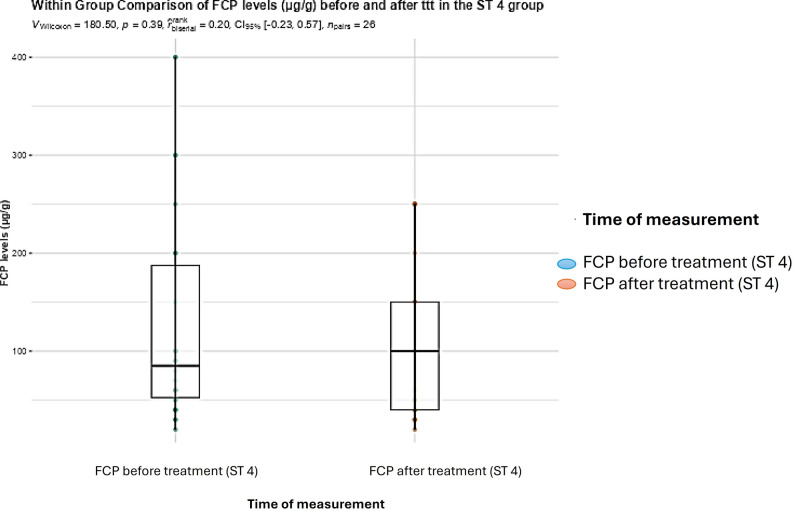
Boxplot showing within group comparison between FCP levels in µg/g (medians and IQRs) before and after treatment with metronidazole in the BC subtype 4 infected group (Wilcoxon rank test; statistical significance at *P* < 0.05)

**Fig. 6:**
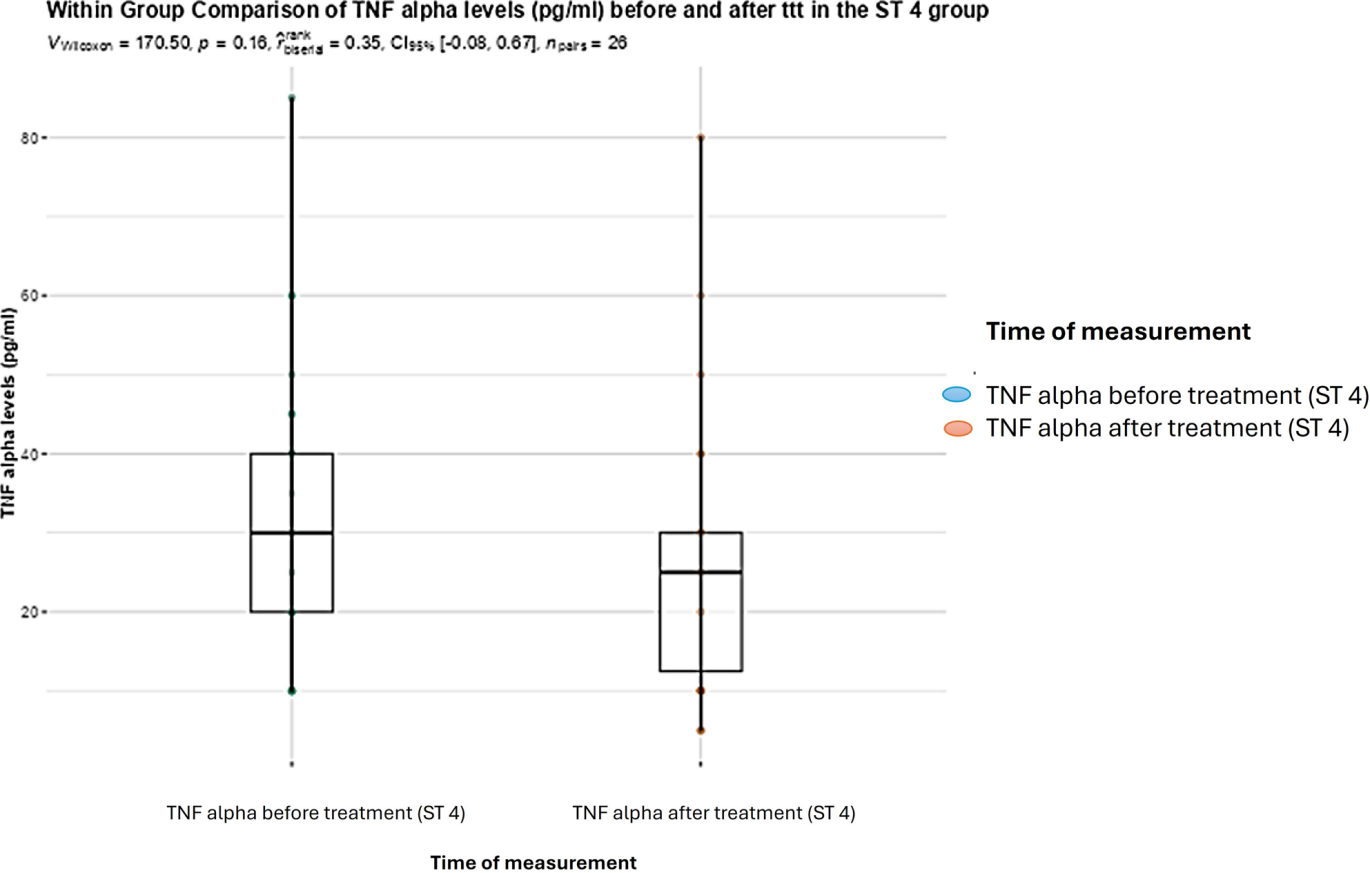
Boxplot showing within group comparison between TNF-α levels in pg/ml (medians and IQRs) before and after treatment with metronidazole in the BC subtype 4 infected group (Wilcoxon rank test; statistical significance at *P* < 0.05)

In BC subtype 4infected patients, no significant difference was found between FCP levels (*P* = 0.39), and TNF-α levels (*P* = 0.16) before and after treatment (Figs. 5 and 6).

FCP and TNF-α levels were numerically higher in patients infected with BC subtype 3 as compared to BC subtype 4, both before and after treatment with metronidazole, however, to no statistical significance. Again, the absence of statistical significance should be interpreted with caution due to the unequal sample sizes in both study groups, especially that the P value is very close to the significant threshold of *P* < 0.05, and that the mean FCP value in the ST3 group is notably higher than that of the ST4 group (Table 2).

**Table 2:** FCP and TNF-α values in both study groups before and after treatment with metronidazole

FCP values (µg/g)			
Time of assessment	Study group		*P* value
	BC subtype 3	BC subtype 4	
*Before treatment with metronidazole*	Mean = 319.0 SD = 303.7 Median = 275.0 IQR = 437.5	Mean = 128 SD = 113.6 Median = 85.0 IQR = 135.0	0.06
*After treatment with metronidazole*	Mean = 177.0 SD = 164.8 Median = 150.0 IQR = 247.5	Mean = 114.0 SD = 84.1 Median = 100.0 IQR = 110.0	0.23

**Fecal TNF-α values (pg/ml)**			
**Time of assessment**	**Study group**		**P value**
	***BC subtype 3***	***BC subtype 4***	
*Before treatment with metronidazole*	Mean = 47.5 SD = 36.2 Median = 44.0 IQR = 33.8	Mean = 33.1 SD = 17.8 Median = 30.0 IQR = 20.0	0.11
*After treatment with metronidazole*	Mean = 31.3 SD = 26.2 Median = 20.0 IQR = 30.0	Mean = 26.2 SD = 17.2 Median = 25.0 IQR = 17.5	0.44

Mann-Whitney U test; Statistical significance at *P* < 0.05

Correlation between both fecal markers was analyzed by Spearman correlation test. No significant correlation was found in both study groups, where the P-value was 0.27 before treatment and 0.39 after treatment in the BC subtype 3 group, and in the BC subtype 4 group the P value was 0.63 before treatment and 0.30 after treatment.

### Fecal biomarkers levels in relation to the parasitological detection of BC in stool samples

Analysis of the association between the microscopic detection of *Blastocystis* stages in stool samples and fecal inflammatory markers was performed. Among patients infected with subtype 3, no significant difference was observed in FCP levels between those with detectable stages and those without. Similarly, TNF-α levels remained consistent regardless of microscopic detection. In the subtype 4 group, FCP levels were numerically higher in patients with detectable stages. However, this difference did not reach statistical significance. It should be noted that the sample size for detectable stages in the ST4 group was limited (*N* = 2), which may compromise the statistical power of this specific comparison (Table 3).

**Table 3:** FCP and TNF-α values in both study groups after treatment with metronidazole in relation to BC presence in stool examination

BC subtype 3			
Fecal biomarker	BC stages in stool samples		*P* value
	*Detected (* *N* * = 18)*	*Not detected (* *N* * = 44)*	
*FCP values (µg/g)*	Mean = 190.6 SD = 140.1 Median = 225.0 IQR = 192.5	Mean = 170.9 SD = 175.1 Median = 100.0 IQR = 260.0	0.43
*TNF-α values (pg/ml)*	Mean = 31.1 SD = 26.3 Median = 25.0 IQR = 27.5	Mean = 31.4 SD = 26.4 Median = 20.0 IQR = 30.0	1.0

**BC subtype 4**			
**Fecal biomarker**	**BC stages in stool samples**		**P value**
	***Detected (N*** ***= 2)***	***Not detected (N = 24)***	
*FCP values (µg/g)*	Mean = 175.0 SD = 35.4 Median = 175.0 IQR = 25	Mean = 108.8 SD = 85.4 Median = 80.0 IQR = 110.0	0.26
*TNF-α values (pg/ml)*	Mean = 30.0 SD = 0.0 Median = 25.0 IQR = 0.0	Mean = 25.8 SD = 17.9 Median = 30.0 IQR = 20.0	0.26

Mann-Whitney U test; Statistical significance at *P* < 0.05

## Discussion

In the current study, symptomatic BC infected patients were recruited to explore the association between BC subtypes and symptomatology of infection, GIT inflammation, and response to treatment with metronidazole. We focused on observing BC subtype-specific associations within a symptomatic cohort without inclusion of a non-infected control, since the objective was not to re-evaluate the pathogenicity of *Blastocystis*, but to explore the possible variabilities among different subtypes in relation to inflammatory outcomes. BC subtype 3 was more prevalent among BC infected patients as compared to subtype 4. Khademvatan et al. [23] identified 5 BC subtypes in 50 samples by targeting SSU rDNA. Among the 5 subtypes (ST1-5) detected, subtype 3 was the most common. In addition, they discovered mixed infections between subtypes 3 and 4, subtypes 1 and 4 and subtypes 1 and 3. In a study from Saudi Arabia, detection of SSU rRNA in BC infected patients revealed the presence of subtypes 1–3, with subtype 3 being the most abundant [24]. They also discovered further genetic diversity within the subtypes by discovering different haplotypes, particularly genotypes 1 and 3. The authors suggested that this genetic diversity may explain the low host specificity of the BC subtypes. Abdo et al. [25] detected SSU rRNA in fecal samples from humans and cattle in Northern Egypt. In human samples subtypes 1–3 were detected, whereas subtypes ST4, ST10 and ST14 were detected in cattle. Several other Egyptian studies from different governorates have also documented the predominance of subtype 3 among BC infected patients [26, 27]. Several techniques have been developed to fine-tune the characterization of BC subtypes. Our choice for PCR-RFLP was guided by literature reports on BC subtypes commonly encountered in Egypt. PCR-RFLP remains an essential tool in clinical settings where rapid subtype identification is required. Our use of RFLP allowed us to categorize the predominant genotype in a large patient group, providing a practical molecular approach for clinical settings. Methods such as Sanger sequencing and high-resolution melting can identify rare BC subtypes, mixed infections, single nucleotide polymorphisms, and specific alleles [28, 29]. Still, PCR-RFLP remains to be a rapid and cost-effective method for the detection of common BC subtypes, and was validated by sequencing by Srichaipon et al. [30].

In the current study, all patients infected with subtype 4 were adults, while 52% of subtype 3 infected patients were children. Reports on the impact of BC subtypes on pediatric GIT disorders are highly variable. In a study conducted on school children in Turkey, Sankur et al. [31] reported that subtypes ST 3, 1, 2, and 7 were detected in their fecal samples. Subtype 3 was found to be the most prevalent one. In a Chinese study, Wang et al. [32] stated similar findings, where subtype 3 was the most common subtype detected among school children, followed by subtypes 1, 7, and 2.

Studies on the association between the genetic diversity of BC and GIT disturbances report a wide array of clinical presentations. Jones et al. [33] analyzed the genetic diversity of *BC spp*. in patients from Oregon, USA, suffering from chronic GIT disturbances. Out of 21 subjects complaining of these symptoms, 9 were found to be positive for BC infection. Again, subtype 3 was the dominant genotype, with only 1 case being infected with subtype 1. Zhao et al. [34] collected fecal samples from 1032 children from a children’s hospital in China, 684 were diarrheic, while 348 were non-diarrheic. Sixty-seven children were found to be infected with BC, 60 of them were complaining of diarrhea. Identification of SSU rRNA revealed the presence of subtypes 1–4, and subtype 7. Subtypes 2, 4 and 7 were found in diarrheic children only, while subtypes 1 and 3 were present in both patient groups. Patients enrolled in the current study were complaining of various GIT symptoms such as vomiting, diarrhea, and constipation. While these manifestations were more frequently observed in the ST3 group, the difference did not reach statistical significance. This lack of significance may be attributed to a limitation in statistical power resulting from the unequal sample sizes between the ST3 and ST4 cohorts. After treatment with metronidazole, both groups showed comparable rates of symptomatic improvement. It is to be noted, however, that metronidazole administration per se can cause side effects such as nausea and vomiting, which may complicate the assessment of clinical improvement [35].

Metronidazole is the conventional antiparasitic drug for the treatment of blastocystosis. It exerts its cytotoxic effect by disrupting parasite DNA. The susceptibility and/or resistance of various BC subtypes to metronidazole is important to be considered in the tailoring of BC management [36]. Roberts et al. [37] conducted an in vitro susceptibility study on the effect of various antimicrobials including metronidazole on BC subtypes 1, 3, 4, and 8. They did not observe a difference between the resistance patterns in the different subtype cultures. Another in vitro study conducted by Rajamanikam et al. [36] assessed the effect of metronidazole on BC subtype 3 as regards parasite multiplication, cysteine protease activity, and promotion of cancer cell proliferation. Cultured BC subtype 3 were not only resistant to treatment, but also showed increased pathogenic potential. This was reflected by the increased BC count, especially the amoebic forms, in addition to the increased protease activity and cancer cell multiplication. In the current study, we have observed a persistence of fecal BC shedding in 29% of ST3 infected patients. In subtype 4 infected patients, fecal parasite clearance was reported in nearly all patients. Again, solid conclusions on subtype-specific resistance patterns to metronidazole cannot be drawn due to the disparity in sample sizes driven by the higher prevalence of BC subtype 3. More expanded studies to include larger cohorts of different BC subtypes are warranted for more solid statistical interpretation of clinical findings.

We have also observed that patients infected with BC subtype 3 showed a marked reduction in both TNF-α and FCP levels after metronidazole therapy. Moreover, baseline FCP levels were numerically higher among subtype 3 patients before the commencement of therapy as compared to subtype 4 patients. Again, the lack of statistical significance may be explained by the presence of outliers and the fewer number of patients infected with subtype 4.

FCP was selected as a marker of intestinal inflammation since its elevation suggests organic rather than functional causes of GIT symptoms [13]. FCP has been detected in fecal samples of patients infected with various parasites such as *Dientamoeba fragilis*, *Giardia lamblia*, *Entamoeba histolytica* and *Blastocystis sp.* [17, 38]. The effect of BC infection on FCP shedding adds to the controversy surrounding the parasite, since both increased and decreased FCP levels were reported in association with BC. Raafat et al. [21] reported increased FCP levels in BC infected patients complaining of GIT manifestations such as abdominal pain, vomiting and diarrhea. In a Mexican study, Nieves-Ramirez et al. [39] focused on the effect of asymptomatic colonization of BC on gut microbiota and the possible association with intestinal inflammation in healthy participants. They found that the asymptomatic infection with BC clearly changed the intestinal microbiotic environment and induced metabolic changes in the carbohydrate, lipid, and protein metabolism. Surprisingly, however, FCP and fecal IgA were decreased in BC-colonized subjects. The authors suggested that the subclinical presence of BC may decrease intestinal inflammation through a direct effect on the intestinal mucosa, or through a possible predatory effect of BC on co-infecting intestinal microbiota. Fecal calprotectin was used by Ibrahim et al. [40] as a marker for the treatment efficacy of nitazoxanide in 12 BC infected children, and 12 *Giardia lamblia* infected ones. They found no significant differences in FCP levels between infected children both pre-and post-treatment.

In the current study, elevated FCP observed among ST 3 infected patients decreased significantly after metronidazole therapy. It is to be noted, however, that metronidazole possesses broad-spectrum activity against various anaerobic bacteria, which precludes the definitive attribution of the reduction of inflammation to the clearance *Blastocystis*, and consequently limits a direct causality claim between the parasite and the inflammatory state. Studies focusing specifically on BC subtype 3 are therefore recommended to explore the implication of this subtype in GIT inflammation and the development of clinical gastrointestinal manifestations. In addition, axenic in vitro studies on the mechanisms of metronidazole and its resistance can provide more conclusive data in absence of confounding co-infection.

Again, reports on the inflammatory potential of BC subtypes are inconsistent. While Rojas-Vela´zquez et al. suggested that BC subtype 3 may protect the intestinal integrity by maintaining eubiosis [6], Hussein et al. reported that subtype 3 along with subtype 1 were highly observed in precancerous colonic polyps in BC infected rats. In fact, the authors reported the presence of 3 genetic variants of subtype 3, namely the wild type, the heterozygous type and the mutant type. The most common type found in the intestinal polyps was the wild variant [41].

TNF-α, along with other pro-inflammatory cytokines, was reported to be up-regulated during blastocystosis, contributing to inflammation and oxidative stress [42]. We have observed a notable decrease in fecal TNF-α after the administration of metronidazole in subtype 3 infected patients. In contrast, the decrease in TNF-α levels after treatment was not as evident in the subtype 4 infected group. Subtype-specific studies reported the association between BC variants and cytokine pattern. Azizian et al. investigated the pro- inflammatory cytokine response in patients with inflammatory bowel syndrome (IBS) infected with BC. They found that patients infected with subtypes 1, 2, and 3 had elevated serum levels of TNF-α and IL-6 [43]. In an in vitro study, Norouzi and colleagues investigated the effect of BC exosomes on cytokine release in a human monocytic leukemia cell line. They demonstrated that cell lines exposed to exosomes derived from BC subtype 4 showed an upregulation of TNF-α and IL-6 levels and a concomitant downregulation of IL-4 and IL-10 levels. IL-10 was also downregulated in response to subtypes 2 and 3 derived exosomes, whereas subtype 3-derived exosomes induced the upregulation of IL-6 [10].

In conclusion, our cohort study showed that BC subtype 3 was the most prevalent subtype among patients reporting gastrointestinal symptoms. The ST3 group exhibited numerically higher inflammatory markers compared to ST4. These observations shed light on the potential link between *Blastocystis* genetic diversity and host intestinal inflammation. The higher trends in fecal biomarker levels in the subtype 3 group warrant more expanded studies for further evidence to support the pathogenic potential of this subtype and its specific contribution to symptomatic disease.

Since this study was designed to be a comparative observational study of a symptomatic cohort of patients within a frame of daily clinical practice, it was faced by several limitations. The investigation of the pathogenicity BC-infection, let al.one that of the individual subtypes, is particularly challenging in clinical settings. Accurate patient reporting of symptoms, especially that metronidazole per se can cause nausea and vomiting, was an important challenging factor in the assessment of clinical improvement in response to therapy. Another limitation was the absence of a control group. While the current study intentionally focused on symptomatic BC infected patients, the absence of a comparison with symptomatic patients free of BC infection complicates the determination of the baseline level of inflammation caused by other GIT diseases. However, the choice of a uniform control group is particularly difficult due to the multiple causes of infectious and non-infectious GIT disorders, and the variable degrees and mechanisms of implicated immunopathological and inflammatory responses. Also, a non-infected control group was not included, since it is certainly challenging to decisively exclude the presence of other causes of GIT infections, due to the large variety of both symptomatic and asymptomatic gut colonizers. In addition, the control of important co-factors of blastocystosis such as viral and bacterial co-infection and their possible effect on FCP and TNF-α are certainly difficult to achieve. Therefore, in vivo and in vitro studies can add to the information obtained from clinical data and provide a deeper analysis of subtype-specific pathogenesis and immune-modulatory effects.

Another limitation was the disparity in sample size between the ST 3 and the ST 4 groups, which reflects the higher prevalence of BC subtype 3 infection among symptomatic BC infected patients. The unequal sample sizes influenced the power of the statistical analysis, despite the notably higher FCP and TNF-α levels observed in the ST 3 group.

Moreover, a technical limitation this study has faced, which is the reliance on PCR-RFLP for genotyping. Since the aim of the study was to adopt a diagnostic approach practical for clinical practice, we have chosen this technique focusing on dominant subtypes reported in studies investigating Egyptian patients. While sufficient for broad categorization, RFLP lacks the sensitivity of Next-Generation Sequencing (NGS) to identify minor-frequency subtypes in a single host. Future research utilizing high-resolution sequencing is necessary to determine if the clinical associations with BC subtypes are driven by specific sub-strains or co-infection with multiple subtypes.

## Supplementary Information

Below is the link to the electronic supplementary material.

Supplementary Material 1

Supplementary Material 2

## Data Availability

All data generated or analyzed during this study are included in this published article.
