# Supplementary material for: Association of Blastocystis species subtypes 3 and 4 with intestinal inflammation and response to metronidazole in symptomatic patients
Source: BMC Infect Dis. 2026 Apr 1;26:744. doi: 10.1186/s12879-026-13047-6 (PMC13064339; doi:10.1186/s12879-026-13047-6)
Supplement: Supplementary file 2 — Supplementary Material 2 [file 12879_2026_13047_MOESM2_ESM.docx]

**Supplementary data**

**Table: Normality check using the Shapiro-Wilk test**

| Normality check using the Shapiro-Wilk test; statistical significance at P<0.01 | | | | | | |
| --- | --- | --- | --- | --- | --- | --- |
|  | **BC subtype** | **FCP before ttt with metronidazole** | **FCP after ttt with metronidazole** | **TNF alpha before ttt** | **TNF alpha after ttt** | **Age** |
| Mean | **ST 3** | 319 | 177 | 47.5 | 31.3 | 29.2 |
|  | **ST 4** | 128 | 114 | 33.1 | 26.2 | 35.7 |
| Standard deviation | **ST 3** | 304 | 165 | 36.2 | 26.2 | 19.0 |
|  | **ST 4** | 114 | 84.1 | 17.8 | 17.2 | 18.2 |
| Shapiro-Wilk W | **ST 3** | 0.852 | 0.857 | 0.823 | 0.792 | 0.888 |
|  | **ST 4** | 0.797 | 0.842 | 0.923 | 0.867 | 0.880 |
| Shapiro-Wilk p | **ST 3** | <.001 | <.001 | <.001 | <.001 | <.001 |
|  | **ST 4** | <.001 | <.001 | 0.052 | 0.003 | 0.006 |
