# Supplementary material for: Association of Blastocystis species subtypes 3 and 4 with intestinal inflammation and response to metronidazole in symptomatic patients
Source: BMC Infect Dis. 2026 Apr 1;26:744. doi: 10.1186/s12879-026-13047-6 (PMC13064339; doi:10.1186/s12879-026-13047-6)
Supplement: Supplementary file 1 — Supplementary Material 1 [file 12879_2026_13047_MOESM1_ESM.pdf]

N-99-2025

Cairo University
Faculty of Medicine
Research Ethics Committee

NOTICE OF APPROVAL

Date: **17-5-2025**

Protocol title: **Exploration of the intraspecies difference between Blastocystis hominis subtypes as regards their relation to intestinal inflammation**

Principal investigator: **Dr. Hala Ramadan &Dr. Noha Mohamed Amin**
Institution or organization: Cairo University

Decision: APPROVAL

Dear Dr. **Hala & Dr. Noha,**

The Research Ethics Committee **(REC)** has reviewed and **approved** the above-mentioned **protocol. You may begin your investigation**. Approval is granted for one year from the date of initial approval. At the end of this period as the principal investigator you will be asked to submit required documents for continuing review.

As principal investigator you will need to:

- Notify the REC Chair immediately after any **serious adverse events** experienced by participants of the investigational study or as reported to you by the sponsor/manufacturer/co- investigators.
- Submit End of trial notification at the end of trial.
- Submit Clinical study report at the end of trial.
- You may not initiate **changes** in approved research protocol without REC review and approval except where necessary to eliminate apparent immediate hazards to the human subjects.
- يحظر سفر أي عينات بشريه من المبحوثين خارج جمهوريه مصر العربية الا بعد موافقة الجهات الامنية .
